# A Review of Biogenic Coalbed Methane Experimental Studies in China

**DOI:** 10.3390/microorganisms11020304

**Published:** 2023-01-24

**Authors:** Run Chen, Yunxia Bao, Yajun Zhang

**Affiliations:** 1 Jiangsu Key Laboratory of Coal-Based Greenhouse Gas Control and Utilization, Carbon Neutrality Institute, CUMT, Xuzhou 221008, China; 2 Key Laboratory of Coalbed Methane Resource & Reservoir Formation History, Ministry of Education, School of Resources and Geosciences, China University of Mining and Technology, Xuzhou 221008, China

**Keywords:** biogenic coalbed methane, microbial community, gas component, biogas reservoirs

## Abstract

Biogenic coalbed methane (CBM) is an important alternative energy that can help achieve carbon neutrality. Accordingly, its exploration and development have become a research hotspot in the field of fossil energy. In this review, the latest detection technologies for and experimental research on biogenic CBM in China in recent decades are summarized. The factors influencing the generation of biogenic CBM and the identification method of biogenic CBM are systematically analyzed. The technologies to detect biogas and the research methods to study microbial diversity are summarized. The literature shows that biogenic CBM is easily produced in the presence of highly abundant organic matter of low maturity, and the organic matter reaching a certain thickness can compensate for the limitation of biogenic CBM gas production due to the small abundance of organic matter to a certain extent. Biogenic CBM production could be increased in an environment with low salinity, medium alkalinity, and rich Fe^2+^ and Ni^2+^ sources. Furthermore, biogenic CBM can be identified by considering three aspects: (1) the presence of gas composition indicators; (2) the content of heavy hydrocarbon; and (3) variation in the abundance of biomarkers. In recent years, research methods to study the microbial community and diversity of CBM-producing environments in China have mainly included 16S rRNA gene library, fluorescence in situ hybridization, and high-throughput sequencing, and the dominant microorganisms have been determined in various basins in China. The results of numerous studies show that the dominant bacterial phyla are commonly Firmicutes and Proteobacteria, while the archaeal fraction mainly includes *Methanoculleus*, *Methanobacterium*, *Methanocorpusculum*, and *Methanothrix*. This review summarizes and discusses the advances in biogenic CBM production and the associated microbial community in order to promote further development of coal biotransformation and CO_2_ bio-utilization to meet energy demands under carbon neutrality.

## 1. Introduction

Fossil fuels are the most important source of energy for human society; however, the burning of coal, in particular, releases large amounts of greenhouse gases that cause climate change [1]. The total energy consumption of standard coal has increased from 517 million tons in 1980 to 4.98 billion tons in 2020 in China [2]. In the foreseeable future, coal remains in the dominant position for energy consumption. Energy consumption has allowed rapid development in China, and it has brought a large number of greenhouse gas emissions. In 2020, about 9.9 billion tons of energy-related CO_2_ was emitted in China, which accounted for 30.9% of the global CO_2_ emissions [3,4]. In 2021, the Chinese government committed to peaking carbon emissions by 2030 and achieving carbon neutrality by 2060. However, this does not mean that fossil fuels will be completely deactivated; instead, two pathways are required: (1) the efficient development and clean utilization of fossil fuel energy; and (2) the isolation, sequestration and utilization of CO_2_.

Coalbed methane (CBM) is an unconventional natural gas that is similar to conventional natural gas in calorific value and CO_2_ emissions, and it has attracted global attention. Biogenic CBM is formed through the synergic degradation of organic matter by the microbial community in a coal seam, and is formed by the microorganisms through both acetic acid fermentation and CO_2_ reduction [5]:

(1) $$\mathrm{acetic}\;\mathrm{acid}\;\mathrm{fermentation}\;{\mathrm{CH}}_{3}\mathrm{COOH}\to {\mathrm{CH}}_{4}+{\mathrm{CO}}_{2}$$

(2) $${\mathrm{CO}}_{2}\;\mathrm{reduction}\;{\mathrm{CO}}_{2}+4H_{2}\to {\mathrm{CH}}_{4}+2H_{2}O$$

The factors influencing the biogenic CBM yield include organic matter abundance, maturity, temperature, pH, Eh, porosity, microbial community, and trace elements. Since the production mechanism of the biogenic CBM was discovered, efforts have been made to increase CBM production through biostimulation and bioaugmentation [6,7]. Biostimulation uses the injection of nutrients to stimulate the indigenous microbial community to degrade organic compounds. Bioaugmentation uses selected allochthonous microorganisms and nutrients to increase methane production [8]. Thus, in this paper, based on the research of biogenic CBM-related fields in China in recent decades, the latest and most widely used research methods in China are summarized. At the same time, the influential factors in the accumulation process of typical biogas reservoirs in China are analyzed, and the identification methods of biogas in coal seams in China are classified. In addition, the unique dominant microorganisms in Chinese coal reservoirs are summarized in this article, pointing out the shortcomings of China in the field of biogenic CBM and proposing future research directions. The objectives of this review are to provide theoretical support for the realistic application of microbially-enhanced CBM production in China and to promote further development of coal bioconversion and CO_2_ bio-utilization in order to meet the energy demand under carbon neutrality.

## 2. Progress in Techniques and Methods

There are numerous techniques and methods to study biogenic CBM, and the main focus of research is on the identification of biogenic CBM and the mechanism of gas production. Identification of biogenic CBM is mainly based on the characteristics of its gas components and the isotope distribution, which are most commonly analyzed through gas chromatography and isotope analysis. The approaches for the analytical assessment of geochemical signatures in natural gas have rapidly developed in recent years, mainly in the form of innovations in geochemical techniques, such as measuring monomeric hydrocarbon hydrogen isotopes, biomarker carbon isotopes, and carbon isotope kinetics, which have led to more effective technical support for the identification of biogenic CBM [9]. The mechanism of biogenic CBM generation has been a research hotspot for Chinese scholars, many of whom have carried out laboratory simulations to study biogenic CBM production and found that the process of microbial degradation to produce biogenic CBM has multiple effects [10]. With the deepening of research, laboratory simulation experiments have revealed a more complete set of research tools for gas, liquid, and solid phase products. Meanwhile, research methods to study coal seam microbial community characteristics have also been actively developed.

### 2.1. Geochemical Technologies

#### 2.1.1. Gas Chromatography

Gas chromatography is the most common means to determine the gaseous composition of biogenic coalbed methane. Due to the limitations of early testing conditions, the utilized gas chromatography techniques had disadvantages, such as cumbersome operation, long analysis time, and large errors in manual injection. With the development of chromatographic techniques, the multidimensional gas chromatography analytical technique, which uses a combination of multiple valves and columns, has emerged [11,12]. This technique can determine the outflow order of biogenic CBM components via the valve opening and closing time, which can facilitate the determination of all components in a sample with one injection. It is easy to operate and largely improves the analysis efficiency, has good repeatability and high accuracy, and is widely used to determine biogenic CBM components [11,13,14].

#### 2.1.2. Isotope Analysis

(1) Hydrogen isotope analysis of monomeric hydrocarbons. This technique mainly uses chromatography mass spectrometry to perform the online analysis of monomeric hydrogen isotopes. It is mainly used to investigate the effect of cracking temperature on the analysis of monomeric hydrocarbon hydrogen isotopes [9,15]. Monomeric hydrogen isotope analysis has made a substantial leap forward in the analysis of hydrogen isotopes, which is important for studying the maturity, origin and formation environment of natural gas, and it also creates the conditions for an in-depth understanding for analyzing the internal microstructure and fractionation process of stable isotopes in nature [13].
(2) Carbon isotope kinetic technique. Carbon isotope kinetic simulation studies can simulate the evolution of stable carbon isotopes and effectively relate this to the sedimentary and thermal history of a basin to dynamically reproduce the formation of natural gas and fractionation of the carbon isotopes [9,16]. Its advantages make it important for identifying gas genesis types and gas sources. Furthermore, it is not only useful for screening primary or secondary gas, but also for predicting the nature and composition of reservoir gas and addressing other related issues.
(3) Biomarker carbon isotope analysis. It was found that the carbon isotopes of biomarkers contain rich biological, geological, and geochemical information, and it is important to study the carbon isotopes within organic compounds [17]. The development of gas/liquid chromatography coupled with stable isotope mass spectrometry has allowed biomarker detection to evolve from simple content analysis to stable isotope analysis of a single compound [18]. Biomarker carbon isotope analysis can establish the connection between biological precursors and their products during diagenesis through structural inheritance and carbon isotope composition characteristics of the monomeric compounds, which can not only explain multiple genesis pathways of the same biomarker compound, but also record the environmental information at the time of organic matter production, as well as the transformation characteristics during degradation, thereby revealing the complex geochemical processes [13,19].

### 2.2. Methods and Means of Products in Biological CBM Simulation Experiments

(1) Gas composition determination. For the characterization of gas components produced during the anaerobic fermentation of coal, the composition of the gas mixture is generally examined using gas chromatography.
(2) Liquid Phase Testing. This technique includes detection of the liquid phase organic and inorganic ions. Gas chromatography-mass spectrometry is generally used to determine the content of volatile organic compounds [20]. For some large-molecule organic compounds that are difficult to volatilize, such as pyruvic acid and polycyclic aromatic hydrocarbons, the more efficient liquid chromatography-mass spectrometer is required [21]. Two methods are generally used for inorganic ion testing: ion chromatography and chemical methods. In addition to detecting anions, ion chromatography is also widely used in the detection of inorganic cations, such as ${\mathrm{SO}}_{4}^{2-}$, ${\mathrm{NO}}_{2}^{-}$ and ${\mathrm{NH}}_{4}^{+}$ [22].
(3) Solid Phase Testing. The solid phase test includes the analysis of components, structure, morphology, functional groups, surface elements, and pores of coal. Physical analysis is often performed on coal according to the national standards for industrial, elemental, specular group reflectance, and maceral components. The structure of coal is generally analyzed using X-ray diffraction with Raman spectroscopy. X-ray diffraction analysis is an effective tool to study the crystal structure and material structure in coal and kerogen [23]. Raman spectroscopy can analyze the changes in single chemical bonds within the structure of coal crystals, as well as the vibrations of groups [24]. Coal morphology and bacteria on the coal surface can be observed by light microscopy, scanning electron microscopy, and atomic force microscopy [9]. Fourier transform infrared spectroscopy is generally used to analyze the structure of coal functional groups during methane production by microbial fermentation. X-ray photoelectron spectroscopy is used to understand the composition and state of elements on the coal surface, which can provide important information about the elemental species, chemical composition, and related electronic structure on the surface of coal samples [25]. The current parameters used to study the coal body pore structure are pore size, pore shape, and pore surface complexity, and the associated testing methods include gas adsorption operation, mercury intrusion porosimetry, microcomputed tomography, and small angle X-ray scattering [26].

### 2.3. Methods and Means of Microbial Community Analysis

**Figure 1:**
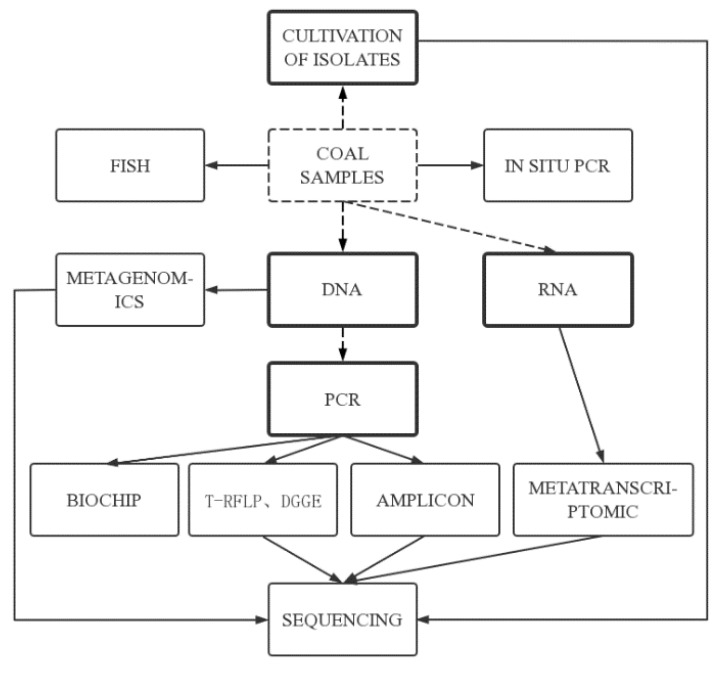
Common molecular approaches to study microbial diversity (Adapted from [30,31,32]). Boxes with bold frames indicate necessary preparatory steps before detection and analysis can be performed; boxes with thin frames represent detection techniques. FISH, fluorescent in situ hybridization; PCR, polymerase chain reaction; T-RFLP, terminal restriction fragment length polymorphism; DGGE, denaturing gradient gel electrophoresis.

The methods for researching microorganisms were developed over 3 stages. The first stage, which occurred prior to the 1970s, involved the development of traditional isolation and cultivation methods, allowing direct observation of colony morphology; however, only a very small number of bacteria with specific physiological functions can be isolated and cultured. The second stage, in the 1970s and 1980s, saw the development of biomarker analyses, which have high sensitivity compared with traditional culture methods and greatly advanced the understanding of microbial community diversity [27]. The third stage, which began in the late 1980s to 1990s, was the development of microbial molecular ecology. This approach was based on the phylogenetic study of 16S rRNA gene sequences (Figure 1), which can more comprehensively reveal the diversity of microorganisms [28,29].

The research on coal seam geological microorganisms in China has mainly focused on the screening and isolation of anaerobic bacteria, enrichment cultures, and experimental simulation of biogenic gas. Initially, the number and distribution of microorganisms in a coal seam were mainly counted by the traditional isolation and culture method: the most probable number counting method [33]. With the progression of experimental techniques in microbiology, scholars in China began to use microbial molecular ecology methods to study the community characteristics and diversity of microorganisms. The main microbial molecular ecology techniques currently applied to study coalbed microorganisms are 16S rRNA gene library analysis, fluorescence in situ hybridization (FISH) and high-throughput sequencing.

16S rRNA gene library analysis, as the main technical means of studying microbial diversity, is widely used in microbial molecular ecology research. Its principle is to extract DNA from microbial samples, obtain 16S rRNA gene sequences by amplification and sequencing of the DNA, and compare the obtained information with sequences in 16S rRNA databases to obtain microbial diversity information. Finger-printing techniques based on 16S rRNA gene sequences, such as denaturing gradient gel electrophoresis and terminal restriction fragment length polymorphism, are widely used to study coalbed microorganisms [27]. The 16S rRNA gene library analysis can detect a variety of bacterial species simultaneously and reflect the abundance of various bacterial species in the bacterial community, but it cannot accurately quantify the number of various bacterial species in the bacterial community [34].

FISH is the use of fluorescently labeled specific oligonucleotide fragments as probes to hybridize with target DNA in the genome to detect the presence and abundance of specific microbial populations. FISH is a molecular technique that does not rely on polymerase chain reaction (PCR) and has the advantage of presenting a clearer picture of the microbial environment and of providing information about some strains that are difficult to cultivate under laboratory conditions. It has been widely used in microbial molecular ecology and environmental microbiology, and has become an important technical tool for microbial community research. The development of this technique has allowed researchers to visualize the symbiotic relationships between microorganisms and promote the development of molecular ecology research of microorganisms [35].

High-throughput sequencing is the most commonly used next-generation sequencing technology, especially in microbial community structure characterization and diversity analysis [36]. Amplicon sequencing, metatranscriptomic sequencing and metagenomics sequencing can meet the needs of the large number and variety of microorganisms in the samples [32]. Amplicon sequencing is a method of amplifying specific regions of microbial community genome based on PCR, and using high-throughput sequencing to study microorganisms composition and function in samples. It has the characteristics of rapidity and low cost in analyzing microbial community [32]. Metatranscriptomics sequencing can correlate microbial community structure and, in situ, function to effectively monitor changes in community structure and function caused by environment [31]. Metagenomics sequencing allows the direct study of genetic material from the microbial community of a sample, independent of culture techniques, to obtain information on a large number of gene fragments within the overall microbial community. The high-throughput sequencing data can be visualized by principal component analysis and Venn diagrams to assess the microbial community structure, and the gene sequences can also be compared with the Kyoto Encyclopedia of Genes and Genomes database to further analyze the relationships between microbial composition and gene function [10].

The development of microbial molecular ecology has facilitated the study of microorganisms in coal seams, and the breadth and depth of microbial diversity analysis has been improved compared to traditional methods. However, different methods have their inherent limitations, such as PCR-based methods, which are prone to bias in microbial understanding due to primer selection and PCR itself, and high-throughput sequencing, which makes it difficult to obtain an accurate map from sequence to function. Therefore, in future research, the choice of method should be based on the specific research content.

## 3. Progress in Influencing Factors and Identification

### 3.1. Biogenic Coalbed Methane Identification

Biogenic CBM has certain similarities and differences compared to conventional natural gas components. The component characteristics and hydrocarbon isotopic composition are often analyzed to identify biogenic CBM; however, due to the phenomenon of isotopic fractionation during the process of formation of some CBM reservoirs, it is necessary to use the degradation characteristics of organic molecules from the coal to identify biogenic CBM when the gas composition cannot be well characterized [37]. Scholars in China have detected the presence of secondary biogenic gas in Liyazhuang in Shanxi, Enhong in Yunnan, Huainan, and Huaibei [38,39,40,41]. Furthermore, about 30 biogenic gas reservoirs have been discovered [42], with proven geological reserves of biogenic gas accounting for about 7% of the total proven natural gas volume [43]. Biogenic CBM can be an important replacement for natural gas and has great potential value. As a clean energy source, it can promote the adjustment of China’s energy structure and help achieve the strategic goal of “carbon peaking and carbon neutrality”. Based on the results of laboratory simulation of biogenic CBM, this paper summarizes the discriminative pathway of biogenic CBM by our scholars into three points: gas composition, isotope composition, and biomarker compounds.

#### 3.1.1. Identification of Gas Composition

The gas composition of biogenic CBM is predominantly characterized by a CH4 content of more than 90%, with little or almost no heavy hydrocarbons [44], which represents a typical dry gas or extra dry gas [38,45]. The proportion of heavy hydrocarbons in the gas is often used as a criterion for biogenic CBM identification, but the upper limit criteria for heavy hydrocarbon content differs between domestic and foreign scholars. Sohoell [46] and Edward et al. [47] suggest that the heavy hydrocarbon content in biogenic gas is generally less than 0.2%, while Whiticar et al. [48] suggest that the heavy hydrocarbon content in biogenic gas should not exceed 1% in general. Domestically, Dai et al. [49] proposed an upper limit of 0.5% for the heavy hydrocarbon content in biogenic gas. Additionally, when studying CBM in a coal seam of Xinji mine in Anhui province, Tao et al. [50] found that the heavy hydrocarbon content was very low as a mixed genesis CBM with an ethane content of 0~0.42% and propane content of 0~0.18%.

At the same time, in order to distinguish biogenic CBM from thermal genesis-generated CBM in low and high coal-grade stages, some of the indicators currently utilized for biogenic CBM discrimination include *φ*(C_1_)/Σ*φ*(C_1_–_5_), *φ*(C_1_)/*φ*(C_2_ + C_3_), and CDMI (CDMI = *φ*(CO_2_)/[*φ*(CO_2_) + *φ*(CH_4_)]) [51]. The *φ*(C_1_)/Σ*φ*(C_1_–_5_) index can distinguish between biogenic and thermally-degraded gases. Biogenic and thermally-cracked gases have dry gas characteristics with *φ*(C_1_)/Σ*φ*(C_1_–_5_) > 0.95, and thermally-degraded gases have *φ*(C_1_)/Σ*φ*(C_1_–_5_) ≤ 0.95. Thermal-cracked gas and biogenic gas can be effectively distinguished by *φ*(C_1_)/*φ*(C_2_ + C_3_) and *δ*^13^C(CH_4_). The value of *φ*(C_1_)/*φ*(C_2_ + C_3_) of biogenic gas is greater than 1000, the value of *φ*(C_1_)/*φ*(C_2_ + C_3_) of thermally degraded gas is less than 100, and the value of *φ*(C_1_)/*φ*(C_2_ + C_3_) of mixed gas is between 100 and 1000. CDMI can discriminate between both inorganic and organic sourced gases. When the CO_2_ concentration in a coal seam reaches 60% or more and the CDMI exceeds 90%, it is judged to be inorganic-formed gas [49]. When the CO_2_ concentration in the coal seam reaches 60% or more and the CDMI exceeds 90%, it is judged as inorganic gas. When the CO_2_ concentration is less than 15%, it is organic genetic gas, among which, the CDMI of the biogenic gas is ≤5%, the CDMI of thermally-degraded gas is ≤90%, and the CDMI of thermally-cracked gas is ≤0.15% [52]. After continuous research by previous authors, the classification of the CBM genesis is summarized (Table 1). Obviously, mastering the geochemical characteristics of each genetic type of CBM can help to better identify biogenic CBM [52].

#### 3.1.2. Identification of Isotopic Composition

**Figure 2:**
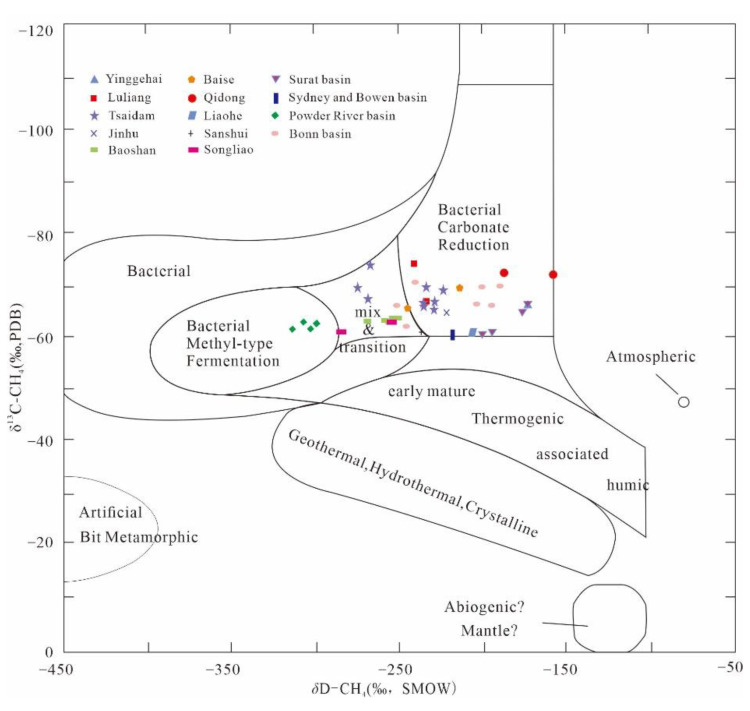
Genetic discrimination of coalbed methane (Adapted from [48,64,65,66,67]).

The carbon and hydrogen isotopic compositions are another way to identify bio-genic CBM. Hu et al. [53] studied the components, including the carbon and hydrogen isotopes, of biogenic gas from five biogenic gas reservoirs in China and found a good negative correlation between the content of iso-alkanes in light hydrocarbon natural gas and *δ*^13^C_1_ values. A *δ*^13^C_1_ < −55‰ is generally chosen as the upper limit standard for biogenic gas during biogenic CBM identification [54,55,56], while the methane *δ*D distribution range is considered to be −400‰ to −150‰. Specifically, acetic acid fermentation genesis methane *δ*^13^C_1_ is distributed from −65‰ to −50‰ and the *δ*D is distributed from −400‰ to −250‰, while CO_2_ reduction genesis methane *δ*^13^C_1_ is distributed from −110‰ to −65‰ and the *δ*D is distributed from −250‰ to −150‰ [56]. Additionally, in the biogas reservoirs found in China, the main formation method of biogenic CBM is CO_2_ reduction, such as in Qidong Basin, Luliang Basin, and Jinhu Basin. A part is biogenic CBM formed by mixed transition, such as in Baoshan Basin, Songliao Basin and Sanshui Basin; another part is biogenic CBM formed by both mixed transition and CO_2_ reduction in the basin, such as in Yinggehai Basin and Qaidam Basin, while there are few biogas reservoirs with acetic acid fermentation genesis, such as the Powder River Basin (Figure 2). It is worth noting that the lower *δ*^13^C_1_ identification value is considered a relative delineation criterion, and the values of biogenic gas *δ*^13^C_1_ in the coal seams of the Xinji, Liyazhuang and Enhong areas of China are significantly less than −55‰ [51].

Meanwhile, many scholars have noticed that the carbon isotopes of CBM are generally lighter than those of conventional CBM [45,57,58,59,60]. Qin et al. [61] indicated that the flow of groundwater leads to methane carbon isotope fractionation, which results in the lightening of methane carbon isotopes in the coal seam. Wang et al. [62] also found that water can fractionate methane carbon isotopes, which become significantly lighter with increasing leaching time. However, further studies are needed to determine whether the dissolution of flowing groundwater causes the lightening of methane carbon isotopes. Different enzymatic reactions are involved in microbial methanogenic pathways with different isotopic fractionation capacities. The distribution of *δ*^13^C-CH_4_ produced by the metabolism of hydrogenotrophic methanogenic archaea ranges from −110‰ to −65‰, while the distribution of *δ*^13^C-CH_4_ produced by the metabolism of acetic acetotrophic methanogenic archaea ranges from −65‰ to −50‰. The range of *δ*D-CH_4_ for CBM is influenced by the maturity of the coal and the hydrogen isotopes in the coalbed water, and its range is relatively large, with *δ*D-CH_4_ generally distributed around −225‰ ± 25‰ [59]. Some scholars also believe that some models or theories cannot account for the lighter carbon isotopes of CBM. Song et al. [63] suggested that the content of CO_2_ in coal seams is low and the carbon isotopes are heavy, and that the methane carbon isotopes become heavier with the desorption–diffusion of CBM, but these models or theories do not explain the general lightening of methane carbon isotopes. The characteristics of hydrocarbon isotopes can be more accurately discerned for CBM in China, but some indicators can only be used as a relative classification standard; thus, the phenomenon of light carbon isotopes of CBM needs further study.

#### 3.1.3. Biomarker Compound Identification

The strength of resistance to organic compound degradation in coal is generally as follows: n-alkanes < isoprenoid alkenes < steranes < patchouli < aromatized steranes < porphyrins [68]. Therefore, biogenic CBM can be identified based on the characteristic changes in gas-producing parent biomarker compounds during the coalification stage. Studies have shown that among saturated hydrocarbons, the degradability of n-alkanes is smaller compared to isoprenoid alkanes, and with increased maturity and burial depth, the odd carbon advantage possessed by n-alkanes gradually disappears and they become preferentially degraded by microorganisms, while isoprenoid alkanes are retained, so the generation of biogenic CBM can be judged based on changes in the relative abundance of the two types [50]. Pan et al. [69] found that after biodegradation of shallow natural gas in the Yinggehai Basin, in addition to a decreased n-alkane concentration, biogenic olefins were detected in gas with weak degradation, which can also be used as a marker for identifying biogenic CBM. It can be seen that the recalcitrant characteristics of the biomarker compounds are important for the identification of biogenic CBM, which can be supplemented if there is uncertainty in the characterization of biogenic CBM biogas components. Thus, the recalcitrant characteristics of biomarker compounds are important for identifying biogenic CBM, which is complementary to the two identification methods of coalbed gas component characteristics and isotope characteristics.

### 3.2. Influencing Factors of Biogenic Gas Generation in Coal Seam

The source rock and reservoir have a profound influence on the gas content and gas generation rate of biogenic CBM. Characteristics such as the difference in organic matter abundance, type, maturity, and deposition environment and burial time will give the biogenic CBM a special state of fugacity. In recent years, scholars in China have conducted in-depth research on the reservoir characteristics of biogenic CBM. As such, this paper summarizes the influence of coal reservoirs on the generation of biogenic CBM in the following aspects.

#### 3.2.1. Effect of Coal Reservoirs on Gas Production

**Figure 3:**
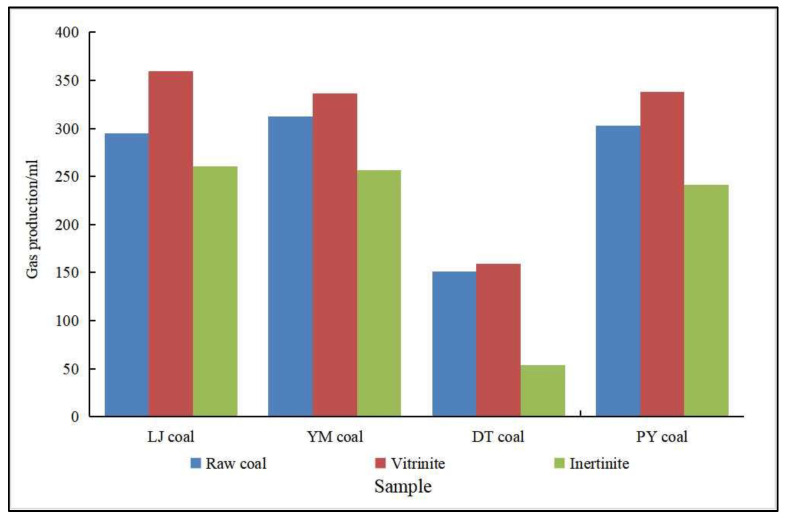
Relationship between maceral and gas production (Adapted from [80,81]). (The coal samples of LiangJia coal mines, YiMa coal mines, DaTong coal mines and PanYi coal mines are abbreviated as LJ coal, YM coal, DT coal, and PY coal, respectively, in Figure).

(1) Organic matter abundance. Organic matter is the material basis for biogenic CBM generation. The higher the abundance of organic matter, the more favorable the biogenic CBM generation. The total organic carbon (TOC) abundance can reflect the organic matter abundance in hydrocarbon source rock to some extent [70] (Table 2). Generally speaking, the higher the TOC, the easier it is to generate biogas. For example, the TOC content of hydrocarbon source rock in the southern Junggar Basin of China is 0.54~1.42%, and the TOC content of hydrocarbon source rock in the Dongying Formation of the Bohai Bay Basin is 0.3~5% [43]. Related studies have shown that atmospheric fields can be formed with a low organic carbon content under high deposition rates and large deposition thicknesses. Huang et al. [71] studied the biogenic gas characteristics of the Yinggehai Basin and showed that the average organic carbon content in source rocks where organic matter is present is about 0.4%, and that the basin is in an active biogenic gas generation stage. The average organic carbon content is 0.3% in the Quaternary lacustrine mudstone of the Qaidam Basin, which also forms an atmospheric field [72]. It has been shown that the thickness of the organic matter in gas source rocks can compensate for the limitation of organic matter abundance to some extent. For example, the existence of thick hydrocarbon source rock in the Norbei basin of the Sanhu depression in China compensates for the low TOC fraction, forming biogenic gas reservoirs with great potential [73,74]. Li [75] studied the abundance of soluble organic matter within hydrocarbon source rocks in the Sanhu area of the Qaidam Basin and found that the abundance of soluble organic matter in the Neoproterozoic–Quaternary strata is much greater than the conventional TOC detection value, which is significant for the evaluation of biogenic CBM resources. Obviously, the higher the organic matter abundance, the more favorable the bio-methane output. It is also worth noting that the evaluation index for organic matter abundance is an important evaluation criterion for biomethane generation, but it is not a decisive factor for evaluating the gas volume of biomethane reservoirs in a region.

(2) Type of organic matter. The organic matter type is closely related to the production of biogenic CBM. Different types of organic matter have different properties and are used by microorganisms with different degrees of ease. Among the organic matter types used to generate biogenic gas, humic parent material in coal is the most easily degraded by microorganisms [76]. Semi-humic and herbaceous humic organic matter can provide the carbohydrates, such as cellulose, hemicellulose, sugars, and starch, needed for microbial metabolism, which promotes biogenic CBM production [54]. Hydrogen-rich, oxygen-rich organic matter also facilitates the production of biogenic CBM. Wang et al. [77] studied the Lu Liang Basin and found rich organic matter, and discovered that the organic matter composition of its Neoproterozoic gas source rock contains more type II casein, which is rich in protein and lipid-like compounds and has great potential for biogenic CBM. Zhang et al. [78] found that the biogenic gas reservoirs of the Sanjiang Basin are also dominated by type II organic matter. Meanwhile, many scholars have studied the hydrocarbon generation capacity of maceral components and found that different maceral components have different hydrocarbon generation capacities. Liu et al. [79] made a systematic study of the gas production characteristics of different maceral and their effect on gas production using thermal simulation experiments, showing that the main gas-producing microfractions were vitrinite and the exinite. Furthermore, with the increase in temperature, the ability of vitrinite to produce methane was higher than that of exinite, while the gas production rate of fusinite was very low. As shown in Figure 3 [80,81], our scholars selected lignite, long flame coal, gas coal, and fat coal produced from LiangJia coal mines, YiMa coal mines, DaTong coal mines, and PanYi coal mines as experimental samples, and found that the main maceral components of gas production were vitrinite/huminite in the biogenic CBM simulation output experiments, while the highest gas production was found in vitrinite/huminite in coal, followed by raw coal, and the lowest production was in exinite/liptinite.

(3) Organic matter maturity. Organic matter maturity determines the biogenic CBM production to some extent (Table 3). It was found that low-rank coal is easier to degrade and produce methane than high-rank coal. The maturity of organic matter in the biogenic gas source rock is generally in the immature-low maturity stage (*R*_o_ < 0.4%) [74,78,82] with a low degree of condensation of organic matter aromatic nuclei. Furthermore, its molecular structure contains a large number of branched chains and oxygen-containing functional groups, and the pore structure of the coal sample is easily changed after biodegradation, which is conducive to biological enzyme activity on macromolecules and, thus, it is easily degraded by microorganisms [83]. Guo et al. [84] found that the organic matter content of low rank coal slurry is higher and that the aliphatic structure, hydroxyl, and amino groups in the coal are easily shed, which promotes microbial degradation. Biogas reservoirs have been formed in basins where the organic matter maturity of the biogas source rock is at the immature–low maturity stage, such as that observed in the Yinggehai Basin [85], the Qidong area of the Yangtze River Delta [86], and the Norbei area [73] in China.

#### 3.2.2. Effect of Coal Reservoir Environment on Gas Production

**Figure 4:**
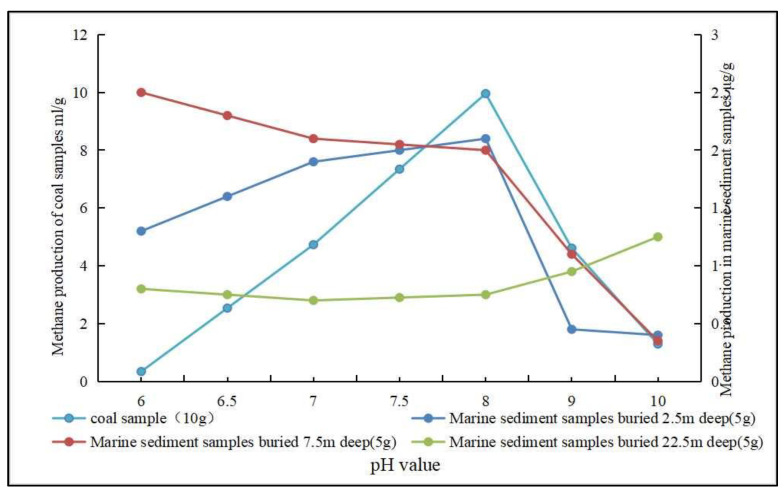
The relationship between pH and gas production (Adapted from [101,102]).

**Figure 5:**
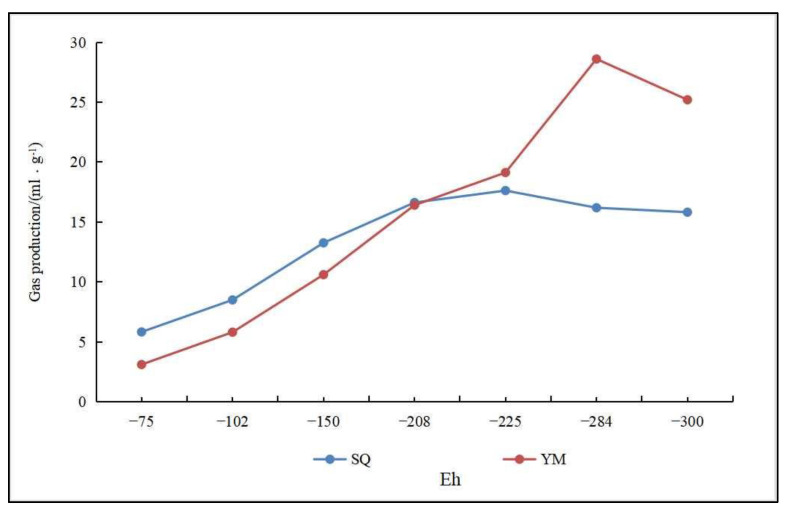
The relationship between Eh and gas production (Adapted from [106,107]). (The coal samples of ShaQu coal mines and YiMa coal mines are abbreviated as SQ and YM, respectively, in Figure).

**Figure 6:**
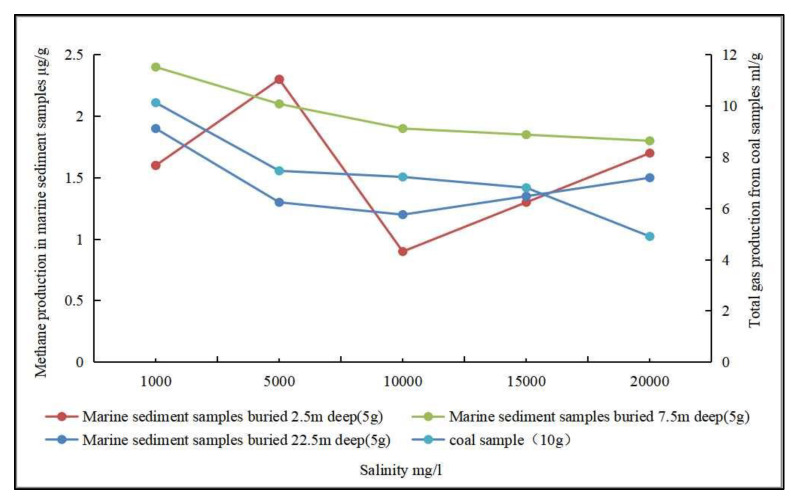
The relationship between salinity and gas production (Adapted from [101,102]).

**Figure 7:**
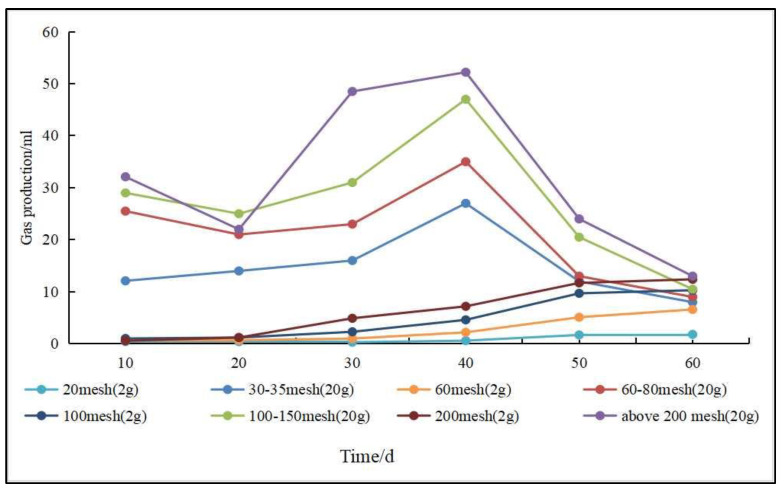
The relationship between particle size and gas production (Adapted from [110,111]).

(1) Temperature. The right temperature will encourage microorganisms to perform at optimum efficiency, maximizing methane production. Through anaerobic coal fermentation experiments at different temperatures, domestic scholars have concluded that the methane yield is largest in the range of 303 K to 328 K, and the biomethane yield increases with increasing temperature within a certain range [88,89,90]. However, further research is needed to study the effect of temperature in a narrower range on the biomethane production. The optimum temperature for anaerobic fermentation of indoor coal is now generally considered to be 308 K [91]. Meanwhile, Tang et al. [92] found that at 338 K, the biomethane production mode was dominated by the acetic acid decomposition type, whereas above 338 K, the biomethane production mode was dominated by the CO_2_ reduction type, indicating that temperature not only affects the activity of methanogenic archaea, but also affects the biomethane output mode.
(2) Trace elements. Trace elements influence the microbial community, and thus methane production during anaerobic metabolism [93,94]. Xia et al. [95] found that the production of biogenic CBM is stimulated by trace elements. For example, the combination of Fe^2+^ and Ni^2+^ has a production-enhancing effect on biogenic CBM, which can shorten the gas production cycle, while the fugacity state of the trace elements in coal shift toward the direction of improving biological effectiveness. Su et al. [96] also found that iron, nickel and cobalt elements affect in situ microorganisms in coal seams, and that iron and nickel elements have a greater impact. Sun et al. [94] investigated the effect of trace elements on the microbial structure in anaerobic fermentation systems and showed that the effect of trace elements on bacterial communities was most prominent for the phyla Bacteroides, thick-walled Bacteroides and Helicobacter, which induced the enrichment of acetic acetotrophic methanogens while promoting the balance between hydrolytic acidification and methanation during anaerobic digestion. Huang et al. [97] found that Fe^2+^ could promote the synthesis of hydrogenase, thereby increasing gas production. The production of CBM can be stimulated by adding trace elements, the effects of which are different for different types of trace elements. The specific reaction mechanism needs to be further discussed.
(3) Ph and oxidation-reduction potential (Eh). The activity, as well as the physiological and biochemical characteristics of microorganisms during anaerobic fermentation, is closely related to the pH, and either too high or too low pH will affect biomethane production [98,99,100]. Biogenic CBM simulation experiments using coal samples as a substrate showed that the CH_4_ output is better at pH 7~8, and both overly-acidic and alkaline environments cause the CH_4_ output to decrease. Biogenic CBM simulation experiments using seafloor sediments as a substrate showed that the effect of pH on CH_4_ output varies with the burial depth of the samples (Figure 4) [101,102]. Jin et al. [103] studied the characteristics of microbial community at different pH and showed that pH affects the activity of coal as well as the microbial metabolic processes. When studying the formation pathway of secondary biogenic gas in Huainan, Ding et al. [104] found that pH within 7.2~7.9 is a suitable range for anaerobic bacteria and methanogenic archaea. It has been suggested that the optimal pH for methanogenesis is within the medium–basic range [105]. The lower Eh are suitable for biogenic CBM generation. Using low-rank coal substrate from ShaQu mine and YiMa mine, it was concluded from simulated biogenic CBM production experiments that a lower Eh makes methanogenic archaea metabolically active, thereby promoting the maximum amount and mass fraction for methane production (Figure 5) [106,107].

(4) Salinity. Salinity has an effect on biogenic CBM production and the activity of methanogenic archaea. Biogenic CBM simulation experiments using low-rank coal as a substrate showed that the activity of methanogenic archaea was restricted with increasing salinity, thereby inhibiting methane gas production, and the corresponding decrease in gas production became obvious with increasing salinity [101]. In contrast, biogenic CBM simulation experiments using seafloor sediments showed that the effect of salinity variation on CH_4_ production was not significant (Figure 6) [102]. Other studies have also shown that increasing salinity causes a decrease in methane production and a lower methane concentration generated by anaerobic coal fermentation, which is not conducive to biogenic CBM production [108,109].

(5) Specific surface area and particle size. The specific surface area and particle size affect biomethane production to some extent (Figure 7). A smaller coal particle size and an larger specific surface area enable microorganisms to be in full contact with the coal surface, which contributes to higher biomethane production [98]. Guo et al. [110] conducted gas production experiments using subbituminous coal and found that gas production increased as the coal particle size decreased. In gas production experiments using lignite of different particle sizes, Wang et al. [111] found that the smaller the particle size, the easier it was to produce methane; however, when the particle size was less than 0.15 mm, the effect of particle size on gas production was no longer obvious.

#### 3.2.3. Deposition Environment and Deposition Time

(1) Sedimentary environment. Some scholars believe that the mode of formation for biogenic CBM is closely related to the sedimentary environment. The formation pathway for biogenic CBM is closely related to the depositional environment. Biomethane in the marine environment is formed mainly through the reduction of CO_2_ [112]. Biomethane in the freshwater environment is formed mainly by acetic acid fermentation [113]. It has also been suggested that the biogenic CBM formation mechanism does not depend entirely on the depositional environment, and that both pathways of formation can exist in both the marine and terrestrial environments [114]. By analyzing the carbon and hydrogen isotopic compositions of 31 gas samples from 10 biogenic gas reservoirs in China, Shen et al. [115] studied their formation pathways and reservoir characteristics and showed that the formation pathway of biogenic CBM under marine conditions was the typical CO_2_ reduction pathway with a heavy hydrogen isotopic composition, but the biogenic gas formed under terrestrial conditions was also mainly the CO_2_ reduction pathway. Therefore, the formation mode of biogas is closely related to, but not entirely dependent on, the sedimentary environment.
(2) Deposition time. Biogenic CBM production is related to the deposition time and, for shallow biogenic CBM, methane increases with deposition time. It has been shown that in well-preserved strata, gas reservoirs with industrial value can be formed with the change in deposition time, even if the organic carbon content is very low [116]. Lin et al. [117,118] studied the Late Quaternary biogenic gas reservoir in the coastal plain of Hangzhou Bay, China, and found that the formation of this gas reservoir did not exceed 12,000 years, and that the total gas volume reached 2445.27 × 10^8^ m^3^. Moreover, the formation in the Dafosi well field in the Ordos Basin, which produces biogenic gas from the coal seam, has undergone a very long period of intermittent deposition, allowing atmospheric precipitation to transport microorganisms into the coal seam and promote the generation of biogenic CBM in the area [119].

### 3.3. Microorganisms

#### 3.3.1. Microbial Characteristics of Coal Reservoirs

The generation of biogenic CBM is inseparable from the activities of the associated microbial community. Similar to a suitable temperature [89], lower redox environment [106], medium alkalinity [101], low mineralization [109], and appropriate trace elements [95], a suitable coal reservoir can provide a good environment for the reproduction of methanogenic archaea, thereby promoting the formation of biogenic CBM [120]. This study shows that the microbial community structure in each coal reservoir has a similar broadness, as well as uniquely dominant microbial groups and abundant taxa. Methanogenic archaea, particularly *Methanoculleus* and *Methanosarcina*, are present in the coal of some coal mining areas in the Ordos Basin, while the bacteria are mainly Actinobacteria and Proteobacteria [91]. Su et al. [96] studied the diversity of microorganisms in coal from 10 representative mining areas of China, showing that microorganisms were more diverse and abundant in areas with known biogenic CBM and that the bacterial communities were mainly dominated by Firmicutes and Proteobacteria, and the prevalent methanogenic archaea were mainly *Methanoculleus*, *Methanobacterium* and *Methanolobus*. Guo et al. [84] analyzed the compositional characteristics of the microbial community in the coal slurry from the Xilingole in Inner Mongolia, the Yanzishan and Malan mines in Shanxi, and the Zhaoguyi mine in Henan province, and found that there was little variability in the composition of different coal slurry microorganisms, with bacteria dominated by *Pseudomonas* and *Macellibacteroides*, and archaea dominated by *Methanocorpusculum* and *Methanothrix*. The study concluded that the main widely-distributed bacteria in coal seams are Proteobacteria, Actinobacteria, Bacteroidetes and Firmicutes, while the archaea are mainly dominated by members of the phylum Euryarchaeota, including Methanococcales, Methanopyrales, Methanobacteriales, Methanosarcinales, Methanomicrobiales, and Methanocellales (Table 4). Recent studies have identified new methanogenic branches, such as Methanomassiliicoccales [121], Methanofastidiosa [122], Methanonatronarchaeia [123], Verstraetearchaeota [124], and Bathyarchaeota [125], but further studies are needed for the detection, isolation and cultivation of novel methanogenic archaea.

Meanwhile, there are differences in the characteristics of microbial community during the four stages of biogenic CBM generation [7]. The main microorganism involved in the hydrolysis phase of polymeric organic matter are *Acinetobacter*, *Lysinibacillus*, *Pseudomonas*, *Bacillus*, *Comamonas* and *Proteiniphilum* [10]. Among them, *Acinetobacter* and *Pseudomonas* are domestics capable of degrading hydrocarbons and many aromatic compounds [142]. *Bacillus* not only enhances the thermal stability of cellulose, but also promotes macromolecule hydrolysis [143]. *Proteiniphilum* is capable of hydrolyzing proteins and lipids [144,145]. Archaea, such as *Methanoculleus* and *Methanobacterium*, are also present during this stage, but they are not very active [10].

The main microorganisms involved in the acid-producing fermentation stage are *Clostridium*, *Macellibacteroides*, *Tissierella*, *Petrimonas, Desulfosporosinus*, and *Desulfitobacterium* [10]. Among them, *Macellibacteroides* ferments large molecules of soluble organic matter generated during the hydrolysis stage that produces small molecules of fatty acids, such as acetic acid, propionic acid, and butyric acid [146]. *Petrimonas* can utilize elemental sulfur and nitrate ions to produce sulfide and ammonia [147]. Both *Desulfosporosinus* and *Desulfitobacterium* are able to decompose propionic acid, benzoic acid, acetic acid, and aromatic compounds [148]. During this process, coenzyme F_420_ activity increases rapidly [149].

The main taxa involved in the hydrogen and acetic acid-producing stage are *Syntrophobacter*, *Sporotomaculum*, *Syntrophomonas*, *Desulfitobacterium*, *Desulfolubous*, and *Enterobacter* [10]. *Syntrophobacter* degrades propionic acid, producing acetic acid and hydrogen [150]. *Sporotomaculum* uses fermented benzoic acid to produce butyric acid, acetic acid, and CO_2_ [151]. Both *Syntrophomonas* are butyric acid-degrading bacteria that oxidize butyric acid to produce hydrogen and acetic acid [152]. *Desulfitobacterium* decomposes propionic acid, benzoic acid, ethanol, and aromatic compounds [148]. *Desulfolubous* degrades propionic acid in the presence of sulfate. The main metabolites of *Enterobacter* include acetic acid, ethanol, and formic acid, as well as small amounts of H_2_ and CO_2_ [153,154]. The hydrotropic methanotrophic *Methanobacterium* is present at this stage, but the methane yield is not significant due to substrate limitation [135].

The main groups of archaea involved in the methanogenic phase are Methanobacteriales, Methanococcales, Methanomicrobiales, Methanosarcinales, Methanopyrales, Methanocellales, and Methanomassiliicoccales [155,156,157]. Almost all methanogens in the orders Methanobacteriales, Methanococcales, Methanomicrobiales, and Methanopyrales are capable of producing methane via the H_2_/CO_2_ pathway [158]. Methanosarcinales is a typical acetic acid-degrading methanogen found in nature [159,160]. *Methanobrevibacter*, *Methanobacterium*, and *Methanocorpusculum* are hydrogenotrophic methanogens that use H_2_ to reduce CO_2_, producing methane [136,161]. *Methanosarcina* and *Methanothrix* are acetic acid trophic methanogenic archaea that use acetic acid as a fermentation substrate [132]. Both *Methanococcus* and *Methanolobus* are methanotrophic methanogenic archaea capable of anaerobic fermentation for methane production by mainly using formic acid, methanol, and methylamines as substrates [132,136,161].

It can be seen that each coal reservoir has its unique dominant and abundant microorganisms, and different dominant microorganisms will be involved in the biogenic CBM formation stage. However, exploration of the physiological and biochemical mechanisms of microbial metabolism during anaerobic coal fermentation for methane production has not yet begun; thus, there is an urgent need to conduct research in the field of metabolomics.

#### 3.3.2. Microbially-Enhanced Biogenic CBM Production

Increased production of biogenic CBM can be achieved by biostimulation and bioaugmentation [162,163]. The addition of chemical substances and organic nutrients, such as trace elements, inorganic minerals, yeast paste, and ethanol, can stimulate the activation of in situ the microbial community of a CBM field, resulting in greater sustained in situ methane production [99,164]. Trace elements, such as Fe, Ni, and Co, are capable of promoting the activity of various enzymes, such as coenzyme F_430_, coenzyme F_420_, and hydrogenase, which have a stimulating effect on biomethane production [95,165,166]. Inorganic minerals such as kaolinite and magnetite also have an effect on biological enzyme activity [167]. The addition of kaolin affects F_420_ enzyme activity, the microbial community structure, and thus the biogenic CBM production process [167]. The addition of nanoscale magnetite [168] promotes the production of acetate and methane, while nanoscale FeCl_2_/FeS_2_ results in the formation of an amorphous substance on the coal surface, both of which result in a more stable anaerobic reaction system and an increased methane yield [169]. Wang et al. [98] stimulated microorganisms to increase methane production by adding nutrients, such as yeast paste. The addition of ethanol can change the structure of coal fermenting microorganisms, thereby changing the way biogenic CBM is produced and also increasing the methane content [170,171]. In a pilot field experiment conducted at the surface of a CBM well in the Zheng1 block of Jincheng (Shanxi, China), in situ injection of nutrient fluid into the coalbed led to an increase in the average gas production from an original 16.81 m^3^/d to 75.13 m^3^/d, thereby achieving increased CBM production [162].

It has been demonstrated that exogenous methanogenic colonies can degrade coal and produce methane, and that heterogenous colonies have a significantly higher gas production efficiency after domestication with coal as the sole carbon source [172,173]. For coal seams with low biomass, the introduction of exogenous high-efficiency methanogenic archaea can increase biomass and optimize coal seam biological populations and stimulate native bacteria while degrading organic matter and increasing methane production [174]. Lin et al. [175] enriched and domesticated anaerobic sludge samples containing anaerobic microorganisms that could utilize coal methanogenic sites, and the results showed that domestication significantly increased the ability of the strains to utilize coal and the amount of gas production increased [175]. Su et al. [176,177,178] found that white-rot fungi were able to degrade coal and that a combined culture of methanogenic archaea and white-rot fungi was the best optimized solution for the biodegradation of lean coal for methane production. Wang [174] screened microbial populations from mangrove sediments for their ability to degrade organic matter for methane production and found that the addition of this microbial population led to increased methane production in a simulated gas production process using lignite as a substrate.

Field experiments studying the addition of nutrients to stimulate indigenous microorganisms and the addition of exogenous methanogenic archaea have demonstrated that both of these methods can achieve enhanced microbial production. However, the problem that the addition of exogenous methanogens to increase CBM production may lead to contamination of the indigenous microorganisms in the coal seam has not been effectively solved, and the understanding of coal biodegradation for methane production is still insufficient, so this method for increased microbial production has not been widely applied. Therefore, in the future, it is necessary to strengthen research on the isolation and metabolic characterization of key microorganisms and to consider the in situ environmental factors in the coal seam, as well as when studying the technology during laboratory simulation of CBM output, which is conducive to advancing the application process of microbially-enhanced CBM production [164].

## 4. Conclusions

The introduction of new technologies and methods has not only led to a deeper understanding of the characteristics of microbial community in coal, but also strengthened the knowledge of the synergistic effects within microbial community. Studying the evolutionary characteristics of organic matter in coal during microbial degradation enriches the basic theory of CBM. Exploring chemical treatment methods for coal has accelerated the course of gas production from the microbial degradation of coal.

(1) An improved understanding of the influencing factors during the process of anaerobic coal fermentation and the mechanism of CBM production has greatly facilitated the process of using geochemical methods to search for natural gas deposits (for example, carbon isotope analysis techniques for biomarker compounds and hydrogen isotope analysis techniques for monomeric hydrocarbons) to better serve the needs of natural gas exploration.
(2) The characteristics of the microbial community in coal and the exploration of synergistic effects within the microbial community have led to the rapid development of microbial production-enhancing technologies, such as bioaugmentation and biostimulation, which not only enrich the current production-enhancing methods for biogenic CBM, but also greatly improves the gas production potential of small and medium-sized gas reservoirs in China. Thus, as an important replacement energy source for natural gas, biogenic CBM is expected to become one of the main clean energy sources for the next stage of national economic development.
(3) Experiments simulating coal gas production demonstrate that chemical pretreatment of coal can effectively depolymerize the complex macromolecules in coal into smaller molecules, making the coal matrix more accessible to microorganisms and, thus, increasing the coal bioconversion rate. The chemical pretreatment of coal provides a new direction to shorten the gas production process of microbial coal degradation, and also facilitates the increased production of biogenic CBM, which can better promote CBM towards a diversified path.
(4) In the context of the “carbon peak, carbon neutrality” strategy, underground coal gasification–carbon capture utilization and storage technologies is one of the essential ways to reduce carbon emissions. Among them, with respect to CBM, in addition to CO_2_ replacement of CBM to achieve increased production of CBM, the injection of CO_2_ into goafs and abandoned mines and the recycling of CO_2_ through CO_2_ methanation can not only achieve the clean utilization of residual coal, but also promote CO_2_ emission reduction, which is expected to achieve the strategic goal of “carbon peak and carbon neutrality” as soon as possible.

At the same time, due to the late start of China’s research in the field of biological CBM production, the microbiological research in China is still limited to the screening and isolation of strains, and studying their characteristics and enzyme activities. Meanwhile, low-temperature and efficient methanogenic strains for CBM production have not been cultivated to adapt to the prevailing coal reservoir temperatures in China, and relevant engineering tests are also lacking. The CO_2_ methanation technology in the field of biogenic CBM is still in the theoretical stage and needs further research. The future research directions for Chinese scholars are likely to focus on the following areas: (1) a study of the physiological and biochemical mechanisms of microbial metabolism during the anaerobic fermentation of coal for methane production; (2) reasonably accessing the best mined and unmined areas for engineering tests for microbial CBM production enhancement; and (3) an in-depth study of the formation mechanism of CO_2_ methanation.

## Figures and Tables

**Table 1:** Classification of coalbed methane genetic types [52].

Genetic Type				Tracer Indicators		*R*_o_/%
				Isotopic Composition *δ*^13^C_1_(PDB), *δ*D_1_(SMOW)	Component Ratio	
Organic genesis	Biogenesis	Protobiogenic gas		*δ*^13^C_1_ < −55‰	*φ*C_1_/*φ*C_1_–_5_ > 0.95	≤0.5
		Secondary biogenic gas		*δ*^13^C_1_ < −55‰ *δ*D_1_: −250‰~−150‰	*φ*C_1_/*φ*C_1_–_5_ > 0.95	0.3~1.5
	Thermo-genesis	Primary thermogenesis	Thermal degradation gas	*δ*^13^C_1_: −46.2‰~−35.1‰ *δ*D_1_: −247.3‰~−225.9‰	*φ*C_1_/*φ*C_1_–_n_: 0.84~0.94 CDMI: 0~90.55%	0.5~2.0
			Thermal cracking gas	*δ*^13^C_1_: −37.5‰~−29.6‰ *δ*D_1_: >−200‰	*φ*C_1_/*φ*C_1+2_ > 0.99 *φ*C_1_/*φ*C_2_ ≥ 3385 CDMI: ≤0.13%	>2~ 2.5
		Secondary thermogenesis		The hydrocarbon isotopes of methane become even lighter	The drying coefficient increased further, but the CO_2_ content increased	>0.5
	Mixed genesis	Gas mixture		*δ*^13^C_1_: −61.3‰~−50.7‰ *δ*D_1_: −242.5‰~−219.4‰ *δ*^13^C_2_: −26.7‰~−15.9‰ Δ*δ*^13^C_C2_–_C1_: 30.7‰~57.4‰	*φ*C_1_/*φ*C_1_–_n_: 0.993~1.0 *φ*C_1_/*φ*C_2_: 188.6~2993.7 *φ*CO_2_ < 2% CDMI: 0.64%~3.06%	>0.5
Inorganic genesis	Inorganic gas				The content of CO_2_ > 60%, CDMI > 90	

**Table 2:** Biologic gas source rock evaluation table [70].

Index	Excellent	Fine	Good	Medium	Poor	Non-Source Rock
TOC/%	>4.0	2.0~4.0	1.0~2.0	0.5~1.0	0.25~0.5	<0.25
chloroform bitumen “A”/ppm	>4000	2000~4000	1000~2000	300~1000	100~300	<100
Organic acid content/%	>2.0	1.0~2.0	0.5~1.0	0.2~0.2	0.1~0.2	<0.1

**Table 3:** Main indicators for classification of mature stages of coal measures source rocks [87].

Index		Immaturity	Low Mature	Mature	High Mature	Over Mature
*R*_o_ (%)		<0.5	0.5~0.7	0.7~1.3	1.3~2.0	>2.0
Buried depth (km)	Present buried depth	<2~3	<2~3.5	<3~5.3	<3.8~6.2	
	Ancient burial depth	<2~3	2~3.5	3~5.3	3.8~6.2	>5~6.2
Paleogeotemperature (K)	Measured ground temperature	<323~363	<333~388	<355~443	<373~467	>403~467
	homogenization temperature of fluid inclusions	<50~90	60~115	85~170	100~194	>130~194
	apatite fission track	<50~90	60~115	85~170	100~194	>130~194
	TTI	<3	3~20	20~160	160~600	>600
Thermal weightlessness of coal (%)		>20				<5
coal volatile (%)		>25~30				<10
Ultimate analysis	H/C atomic ratio	0.75~0.85				<0.6
	O/C atomic ratio	<0.2~0.25				<0.05
Exinoid group	thermal alteration index	<2.5	2.5~3.5	3.5~4.5	>4.5	5
	color	faint yellow -yellow	yellow-yellowish-brown	Yellowish brown-brown	Dark brown-brownish black	black
Pyrogenation	T_max_ (K)	<703	703~710	710~748	743~813	>773
	IH (mg/g)	150~250				<0.6

**Table 4:** Bacterial and archaeal genera found in microbial consortia.

	Genus	Substrate	Ref:
Bacteria			
Proteobacteria	*Syntrophus*	long-chain fatty acid; aromatics; long chain alkane	[126]
	*Rheinheimera*		[127]
	*Pseudomonas*		[128]
	*Acinetobacter*		[129]
	*Arcobacter*	Citrate	[129]
	*Ferribacterium*	aromatic hydrocarbon; naphthalene	[127]
	*Desulfofustis*		[130]
	*Desulfonema*		
	*Aquamicrobium*	polycyclic aromatic hydrocarbons	[131]
	*Achromobacter*	saturation; aromatics; organic acids; amino acids; carbohydrates	[131]
	*Advenella*		
	*Comamonas*		
	*Thauera*		
	*Desulfovibrio*	carbohydrates; hydrocarbon; organic acids	[131]
	*Citrobacter*	hydrocarbon	[131]
	*Stenotrophomonas*		
Actinobacteria	*Gordonia*	dibenzothiophene	[96]
	*Mycobacterium*		
	*Rhodococcus*		[129]
	*Brevundimonas*		[129]
	*Cellulomonas*	cellulolytic; poor water soluble organic compounds	[131]
Bacteroidetes	*Bacteroides*	carbohydrate; saccharides	[129]
	*Petrimonas*	organic acids and polymers	[131]
	*Prolixibacter*	VFA	[96]
	*Proteiniphilum*	protein; alcohols; saccharides	[96]
	*Sediminibacterium*		
	*Macellibacteroides*		
Firmicutes	*Clostridium*	starch; cellulose; chitin; xylan	[132]
	*Acidoaminococcus*		[133]
	*Ruminococus*		[130]
	*Sporomusa*		[128]
	*Tissierella*	aromatics; amino acid	[96]
	*Tyzzerella*	macromolecular	[131]
	*Lachnoclostridium*	cellulolytic	[131]
	*Anaerofilum*		
	*Syntrophomonas*		[134]
	*Acetonema*		[131]
	*Dehalobacter*	chlorinated aliphatic; aromatic compounds	[131]
	*Desulfitobacterium*		[131]
Synergistetes	*Aminobacterium*	amino acids	[131]
Methanogen			
Hydrogenotrophic	*Methanocorpusculum*	H_2_/CO_2_	[135]
	*Methnolinea* *Methanoregula*		[136]
	*Methanosphaerula*		
	*Methanothermus*		
	*Methanocaldococcus*		
	*Methanogenium*		
	*Methanolacinia*		
	*Methanopyrus*		
	*Methanotorris*		
	*Methanofollis*		[131]
	*Methanocalculus*		
	*Methanocella*		[137]
	*Methanoculleus*		[96]
	*Methanobacterium*		
	*Methanothermobacter*		
	*Methanothermococcus*		[138]
	*Methanoplanus*		[139]
Aceticlasitic	*Methanococcus*	acetic acid H_2_/CO_2_	[138]
	*Methanosaeta*		[134]
	*Methanothrix*		[96]
	*Methanobrevibacter*		
	*Methanomicrobium*		
	*Methanolinea*		
Methylotrophic	*Methanosphaera*	Methylamine; formic acid; methanol	[136]
	*Methanomassiliicoccus*		
	*Methermicoccus*		
	*Halomethanococcus*		
	*Methanosalsum*		
	*Methanohalobium*		
	*Methanohalophilus*		
	*Methanococcoides*		
	*Methanomethylovorans*		[96]
	*Methanolobus*		
	*Methanosarcina*		[134]
	*Methanimicrococcus*		[140]
	*Methanofastidiosa*		[141]

## Data Availability

Not applicable.
